# Supplementary material for: A Likelihood Approach for Real-Time Calibration of Stochastic Compartmental Epidemic Models
Source: PLoS Comput Biol. 2017 Jan 17;13(1):e1005257. doi: 10.1371/journal.pcbi.1005257 (PMC5240920; doi:10.1371/journal.pcbi.1005257)
Supplement: S1 File — (TAR.GZ) [file pcbi.1005257.s014.tar.gz › HSPH_Online-SI-Revision/IPoi09/n1-mild/n1-mild_BENCH2_summary.pdf]

peak −8	3w spec	3w cum	5w spec	5w cum	end	3w spec LOG	3w cum LOG	5w spec LOG	5w cum LOG	end LOG
mean	223.7	184.9	161.	164.7	74.3	0.7	0.6	0.8	0.7	0.7
median	137.3	124.3	114.7	132.3	68.9	0.6	0.6	0.7	0.6	0.6
quantile .1	75.4	74.4	56.	68.7	47.7	0.4	0.3	0.3	0.3	0.2
quantile .9	408.5	378.1	272.2	364.4	105.2	1.	1.	1.3	0.3	1.4
46	47	43	46	38						

peak −4	3w spec	3w cum	5w spec	5w cum	end	3w spec LOG	3w cum LOG	5w spec LOG	5w cum LOG	end LOG
mean	102.3	103.7	104.2	83.4	71.7	0.5	0.4	0.6	0.4	0.5
median	83.2	87.5	82.	71.6	69.1	0.4	0.4	0.4	0.3	0.3
quantile .1	42.1	47.6	50.	39.9	40.1	0.2	0.2	0.2	0.2	0.2
quantile .9	177.2	184.7	156.4	149.4	113.6	0.9	0.6	1.3	0.2	0.9
41	42	37	41	28						

peak	3w spec	3w cum	5w spec	5w cum	end	3w spec LOG	3w cum LOG	5w spec LOG	5w cum LOG	end LOG
mean	136.5	111.4	1375.3	115.1	103.7	0.4	0.3	0.5	0.4	0.3
median	102.3	105.1	119.6	107.9	77.5	0.4	0.3	0.4	0.3	0.3
quantile .1	56.1	55.5	56.1	58.3	42.9	0.2	0.2	0.2	0.2	0.2
quantile .9	244.3	174.2	238.4	177.	187.9	0.7	0.5	0.8	0.2	0.5
27	27	26	22	29						

peak +4	3w spec	3w cum	5w spec	5w cum	end	3w spec LOG	3w cum LOG	5w spec LOG	5w cum LOG	end LOG
mean	652.	2002.7	1441.	2628.1	3237.	0.5	0.4	0.6	0.4	0.5
median	107.1	108.1	115.5	100.5	82.9	0.4	0.3	0.5	0.3	0.3
quantile .1	53.2	56.4	60.6	55.2	48.1	0.2	0.2	0.3	0.2	0.2
quantile .9	395.1	243.6	1627.1	268.7	359.2	0.7	0.5	1.4	0.2	0.8
32	33	33	32	28						

peak +8	3w spec	3w cum	5w spec	5w cum	end	3w spec LOG	3w cum LOG	5w spec LOG	5w cum LOG	end LOG
mean	1327.9	1525.8	1309.	1949.8	2465.2	0.6	0.5	0.7	0.6	0.6
median	110.9	123.6	131.4	101.7	88.3	0.4	0.4	0.6	0.4	0.4
quantile .1	58.4	67.1	66.2	63.	52.9	0.2	0.2	0.3	0.2	0.2
quantile .9	4941.9	821.8	3615.4	1123.7	1717.3	1.4	0.8	1.2	0.2	1.3
41	37	46	39	36	{}	{}	{}	{}	{}	{}
